# Mass spectrometry-based proteomic strategy for ecchymotic skin examination in forensic pathology

**DOI:** 10.1038/s41598-023-32520-9

**Published:** 2023-04-14

**Authors:** Lorenzo Toma, Giulia Vignali, Elisa Maffioli, Stefano Tambuzzi, Roberta Giaccari, Monica Mattarozzi, Simona Nonnis, Marco Milioli, Lorenzo Franceschetti, Gianluca Paredi, Armando Negri, Benedetta Riccardi, Cristina Cattaneo, Maria Careri, Gabriella Tedeschi, Stefano Bruno

**Affiliations:** 1 Department of Chemistry, Life Sciences and Environmental Sustainability, University of Parma, 43124 Parma, Italy; 2 Institute of Legal Medicine, Department of Biomedical Sciences for Health, University of Milan, 20133 Milan, Italy; 3 Department of Veterinary Medicine and Animal Science, University of Milan, 26900 Lodi, Italy; 4 Food and Drug Department, University of Parma, 43124 Parma, Italy; 5 CRC Innovation for Well-Being and Environment (I-WE), University of Milan, 20133 Milan, Italy; 6 Department of Pharmacokinetic, Biochemistry and Metabolism, Global Research and Preclinical Development, Chiesi Farmaceutici Spa, 43122 Parma, Italy

**Keywords:** Biomarkers, Proteome

## Abstract

Mass spectrometry (MS)-based proteomics has recently attracted the attention from forensic pathologists. This work is the first report of the development of a shotgun bottom-up proteomic approach based on rapid protein extraction and nano-liquid chromatography/high-resolution mass spectrometry applied to full-thickness human skin for the differential analysis of normal and ecchymotic tissues to identify new biomarkers for bruise characterization and dating. We identified around 2000 proteins from each pooled extract. The method showed excellent precision on independent replicates, with Pearson correlation coefficients always higher than 95%. Glycophorin A, a known biomarker of vital wounds from immunochemical studies, was identified only in ecchymotic tissues, as confirmed by Western blotting analysis. This finding suggests that this protein can be used as a MS-detectable biomarker of wound vitality. By focusing on skin samples from individuals with known wound dating, besides Glycophorin A, other proteins differentially expressed in ecchymotic samples and dependant on wound age were identified, although further analysis on larger datasets are needed to validate these findings. This study paves the way for an in-depth investigation of the potential of MS-based techniques for wound examination in forensic pathology, overcoming the limitations of immunochemical assays.

## Introduction

Among the recurring tasks in forensic pathology, the examination and characterization of wounds are particularly relevant, especially in cases of violent death. Skin wounds—which include incisions, burns, and contusions—are among the most common in forensic practice^1,2^. If the individual is alive at the time of the injury, blood pressure produces an extravascular collection of blood beneath the intact epidermis—known as hemorrhage or bruise—following arterial, capillary, or venous damage. Bruises are mainly caused by blunt forces directed to the skin surface^3^.

The distinction between vital skin wounds—i.e., wounds that predate the death event—and lesions that occur after death is a crucial goal in forensic pathology^4^. In non-putrefied corpses, the evidence of hemorrhagic tissue infiltration in the skin lesion is commonly considered a macroscopic sign of vitality, whereas its absence indicates that the lesion is likely post-mortem^5^. However, hemorrhages can be confused with livor mortis, and pre-existing contusions can be accentuated by death since hemoglobin filters through the tissues^6–8^. To identify vital skin wounds, conventional histological evaluation is routinely used, whereas immunohistochemistry and immunofluorescence methods are only applied in more complex cases. However, neither technique is useful when the injury occurs immediately before death. Furthermore, no reliable method is available to identify vital wounds in decomposed bodies, where the macroscopic appearance of the skin is completely subverted^9^.

Once the vitality of the wound has been defined, the assessment of the wound age—i.e., the time between the trauma and death—is fundamental information for establishing the causal relationship between the two events^1,10–12^. In the last decades, progress has been made in wound-age estimation, although no reproducible system or model has been proposed as definitive^1,13^.

In this context, proteomics could represent a promising approach for wound examination to overcome the known limitations of immunohistochemistry, i.e., low accuracy, low reproducibility and operator bias. In forensic investigation and legal medicine, proteomics is in its infancy, but it can be a confirmatory and orthogonal technique to well-established DNA-based methods, as well as an additional strategy for revealing useful information and facing new analytical challenges^14^. The few attempts at MS-proteomic analysis for the investigation of wound vitality have used a low-throughput approach based on two-dimensional gel electrophoresis separation followed by matrix-assisted laser desorption/ionization-time of flight-mass spectrometry (MALDI-TOF). Tarran et al.^15^ studied excision wounds on full-thickness skin in a rat model: twenty-six spots from the 2D-PAGE gel were identified by MALDI-TOF analysis, highlighting hemoglobin as the protein most subject to changes. In the same year, Pollins et al.^16^ performed 2D difference gel electrophoresis (2D-DIGE) on a protein extract from normal human partial-thickness skin and burn wounds; forty-six proteins were identified by MALDI-TOF-MS, with some potentially involved in healing.

Advances in liquid chromatography-mass spectrometry (MS)-based proteomics have recently attracted the attention of forensic scientists and pathologists to answer complex forensic questions and strengthen scientific evidence for legal cases^14,17–20^. However, shotgun proteomics with high-throughput LC-MS/MS analysis of skin samples is still an unexplored approach for wound characterization and dating, which are still mostly assessed through conventional histological evaluations. High throughput MS-based proteomic strategies can therefore be exploited to develop reliable analytical approaches to overcome the limitations associated with traditional methods for wound examination and dating.

In the development of a bottom-up LC-MS-based proteomic method, a fundamental step is sample treatment: although a standardized workflow involves protein extraction, proteolytic digestion and sample clean-up, it needs to be tailored to the specific application in terms of matrix type, sample size, protein amount and solubility, co-extraction of interfering compounds, instrumental analysis, etc.^21–24^. The sample treatment protocol has to ensure not only high extraction and purification efficiency and high protein coverage, but also good precision and, possibly, high-throughput performance. To the best of our knowledge, Bliss et al.^23^ is the only study developing a multi-step sample preparation protocol and on-line 2D-LC-MS platform for shotgun proteomic investigation on full-thickness skin; it is only aimed at maximizing proteome coverage compared to a standard laboratory protocol, without a definite medical or forensic goal. It should be noted that the devised procedure involves a time-consuming tissue cryosectioning previous to mechanical homogenization and a chromatographic separation lasting 8 h to enhance protein coverage. The authors identified more than 2000 proteins, but no results about precision of the analytical strategy were shown.

The present study represents the first time that a LC-HRMS-based method was devised for shotgun proteomics on autoptic full-thickness human skin with particular attention on the development of a simple and high-throughput sample treatment protocol. The method was then applied for differential analysis between normal and ecchymotic tissues to gain insights into the proteomic profiles of bruises in search for markers of wound vitality and age.

## Materials and methods

### Chemicals

Deionized water was obtained by Milli-Q Element water-purification system (Millipore, Bedford, MA, USA). Urea was purchased from VWR International (Milan, Italy). Hexane (≥ 99%), trypsin sequence-grade, 1,4-dithioerythritol (DTE), iodoacetamide (IAA), acetonitrile and formic acid were purchased from Sigma Aldrich (Milan, Italy).

### Sample collection

Full-thickness human skin samples, comprising epidermis, dermis, and subcutaneous tissue, were collected at the Institute of Legal Medicine in Milano—where about 700 autopsies are performed every year—in accordance with article 41 of the Italian National Police Mortuary Regulation (September 10, 1990; n° 285) and the Regio decreto (1933 n. 1592, art 32). Tissues were taken in order to answer judicial issues raised by the prosecution performing further analysis. Sample collection was performed in accordance with the Declaration of Helsinki.

Samples were taken only from people younger than 65, in good health and in traumatic death (fall from height, traffic accidents, homicides). For each case, both ecchymosis (E) and adjacent normal tissue (N, control sample) were collected. Excision of small fragments of cadaveric skin was performed using a scalpel. A number of 18 couples of skin samples were investigated for cases with unknown wound dating. For cases 1–12, the excised fragments of ecchymotic and normal tissue were split in two and each part was subjected to the same protein extraction procedure (Table 1).

**Table 1:**
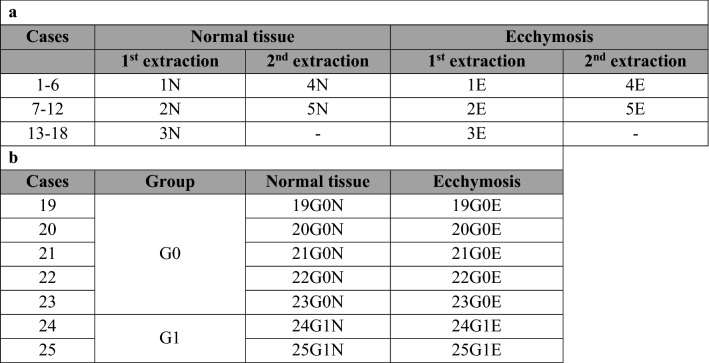
Overview of the analyzed samples.

For each case, both ecchymosis (E) and adjacent normal tissue (N, control sample) were collected. (a) List of 18 couples of skin samples for cases with unknown wound dating. The ecchymotic and normal full thickness skin samples from 6 cases were pooled as indicated; (b) List of 7 couples of skin samples for cases with known wound dating, grouped in two classes: Group G0 (death within 1 h after trauma) and Group  G1 (death within 1–12 h after trauma).

In addition, 7 cases having known age of ecchymosis wound were also analyzed; in particular, these samples were grouped in two classes: 5 belonged to Group G0 (death within 1 h after trauma) and 2 to Group G1 (death within 1–12 h after trauma) (Table 1). Skin samples were stored in plastic tubes at -80 °C until analysis.

### Sample treatment

Ecchymotic and normal skin samples were treated in series. In particular, around 100 mg of skin were transferred into a clean tube for the defatting step: 500 µL of hexane were added and the sample was vortexed for 5 min. Then, after complete hexane evaporation, the skin sample was weighted to assess weight reduction due to loss of fat components. The sample was pulverized under liquid nitrogen by using a homemade stainless steel closed mortar grinder. The pulverized sample was put into a 2 mL reinforced tubes prefilled with inert 2.8 mm stainless beads (MK28-R, Precellys Lysing Kit) and 1 mL of urea 8 M aqueous solution was added. Homogenization was carried out by a Precellys Evolution tissue homogenizer (Bertin Instruments) equipped with cooling unit (Cryolys Evolution), performing 5 cycles of 30 s at 8500 rpm, with 40 s break between cycles. Up to 24 samples can be processed in parallel. Then, the homogenate was transferred into a 1.5 mL centrifuge tube, it was centrifuged at 13,200 rpm for 15 min at 4 °C and the supernatant was retained.

The concentration of total extracted protein was then quantified by absorption spectroscopy at 280 nm using a Cary 400 spectrophotometer (Variant) and a 150 µL quartz cuvette. Two-way analysis of variance with interaction (two-way ANOVA) was carried out with Statgraphics Centurion software.

The ecchymotic and normal full-thickness skin samples, collected from cases with unknown wound dating, were pooled based on protein concentration to overcome individual variability, so that each pooled sample contributes the same concentration.

In details, five different pools were made for both ecchymotic (1E, 2E, 3E, 4E and 5E) and normal skin (1N, 2N, 3N, 4N and 5N), each one with 100 µg of proteins extracted from six cases. Samples 4 and 5 were made up pooling together the proteins obtained from the second extraction of cases 1–12, as detailed in Table 1.

The protein content of each pool was quantified using Bradford assay.

Concerning samples collected from cases having known age of wound, they were analyzed individually.

For proteomic analysis prior to proteolysis, all samples were reduced with 13 mM DTE (15 min at 50 °C), alkylated with 26 mM IAA (30 min at RT, in the dark) and quenched with 1 mM aqueous methylamine^25^.

The protein mixtures were diluted in 20 mM ammonium bicarbonate pH 8, and digested overnight with sequence-grade trypsin enzyme, at 37 °C using a protein:trypsin ratio of 20:1^26^.

The digestion was blocked by acidification of the samples to inactivate the enzyme.

### Nano-liquid chromatography/high-resolution mass spectrometry analysis

Before label-free shotgun MS analysis, the proteolytic digests were desalted using Zip-Tip C18 as described in Vernocchi et al.^27^.

All samples were analyzed using a Dionex Ultimate 3000 nano-LC system (Sunnyvale CA, USA) connected to Orbitrap Fusion™ Tribrid™ Mass Spectrometer (Thermo Scientific, Bremen, Germany) equipped with nano electrospray ion source. Peptide mixtures were pre-concentrated onto an Acclaim PepMap 100–100 μm x 2 cm C18 (Thermo Scientific) and separated on EASY-Spray column ES802A, 15 cm × 75 μm ID packed with Thermo Scientific Acclaim PepMap RSLC C18, 3 μm, 100 Å using mobile phase A (0.1% formic acid in water) and mobile phase B (0.1% formic acid in acetonitrile 20/80, v/v) at a flow rate of 0.300 μL/min. The temperature was set to 35 °C and the samples were injected in duplicates.

The MS was operated in positive and data-dependent acquisition mode to automatically alternate between a full scan (m/z 375–1500) in the Orbitrap, at resolution 120,000 (at 200 m*/z*), cycle time 3 s between master scans, and subsequent HCD MS/MS with collision energy set at 35 eV.

The acquired raw files were subjected to data analysis using MaxQuant software (version 1.6.0.1, https://maxquant.org/). The searches were performed with the built-in Andromeda search engine against the reference *Homo sapiens* proteome (updated on 04/2021; 77,046 sequences), from Uniprot (https://www.uniprot.org/proteomes). The following settings were selected for analysis: strict trypsin specificity allowing up to two missed cleavages, the minimum peptide length was seven amino acids, carbamidomethylation of cysteine was set as a fixed modification. Oxidation of methionine, deamidation of asparagine and glutamine and acetylation of the protein N-terminal were set as variable modifications. Only peptides containing at least seven amino acids were accepted, and a False Discovery Rate (FDR) of 0.01 was applied to both peptides and proteins. 'Match between runs' was enabled with a match time window of 0.7 min and an alignment time window of 20 min. Relative, label-free quantification (LFQ) of proteins using a minimum ratio count of one, was performed in MaxQuant, as described previously^28^.

### Bioinformatics

The protein groups identified by MaxQuant were analyzed by the Perseus software (version 1.5.5.3)^28^. Hits to the reverse database were eliminated and the LFQ intensities were converted to a log scale (log_2_). Only proteins present and quantified in at least one out of two repeats were considered as positively identified in a sample. A Student’s t-test (FDR ≤ 0.05) was carried out to identify proteins differentially present among the different conditions. Proteins were considered to be differentially present if they were present only in one condition or showed significant t-test difference (FDR ≤ 0.05). The precision of the protein extraction was determined comparing dataset deriving from the first and the second protein extraction available for cases 1–12, 1E versus 4E, 1N versus 4N, 2E versus 5E and 2N versus 5N, in terms of number of identified proteins and LFQ intensity of the signals. The Pearson correlation coefficient values were calculated using the log_2_ LFQ intensity for the comparisons reported above.

### Western Blot analysis

The proteins extract pools used for the MS experiments were precipitated in 10% trichloroacetic acid (TCA) to remove urea, re-solubilized in sample buffer (2% sodium dodecyl sulfate, 5% 2-mercaptoethanol, 10% glycerol, and 0.05% bromophenol blue in 0.0625 M Tris–HCl, pH 6.8), and separated by 12% SDS polyacrylamide electrophoresis (20–44 µg of total protein content per lane depending on the antigen), along with a pre-stained protein marker (PanReac, Applichem). Proteins were then transferred to nitrocellulose membranes (Amersham ™ Protran ™, GE Healthcare) by standard methods. The membranes were incubated overnight at 4 °C with 3% skimmed milk (Millipore). They were then incubated with the primary antibodies for either 2 h at room temperature (for the anti-Glycophorin A antibody) or for 12 h at 4 °C (for the anti-GAPDH antibody). Finally, the membranes were incubated for 2 h at room temperature with an HRP-conjugated anti-Rabbit IgG (Sigma-Aldrich, A0545, 1:1500). Immunoblots were visualized by chemiluminescence with the AppliChem HRP substrate (A3417, 1200A-B) using Chemidoc (Bio-Rad). Protein densitometry was performed using the Image Lab 6.0.1 software (Bio-Rad). GAPDH was used as an internal control to verify equal protein loading. The primary antibodies were rabbit anti-CD235a (Glycophorin A) (Thermo Fisher Scientific, PA5-85,882, 1:1000) and rabbit anti-GAPDH (Sigma Aldrich, HPA040067, 1:2500).

### Ethics approval

This article does not contain any studies with human living participants or animals performed by any of the authors. Informed consent for the skin samples was not needed (Police Mortuary Regulations, DPR 09/10/1990 n° 285, art. 41). Data were acquired as part of a forensic judicial investigation and in accordance to Italian Police Mortuary Regulation (Mortuary Police Regulations, Presidential Decree 285, September 10, 1990). In accordance with Italian law, ethical approval is not required in these cases, however the anonymity of the subjects must be guaranteed. Moreover, all the studies conducted followed the guidelines provided by Legislation and the National Bioethical Committee and guidelines by Helsinki Declaration.

## Results and discussion

### Development of the protein extraction procedure

**Figure 1:**
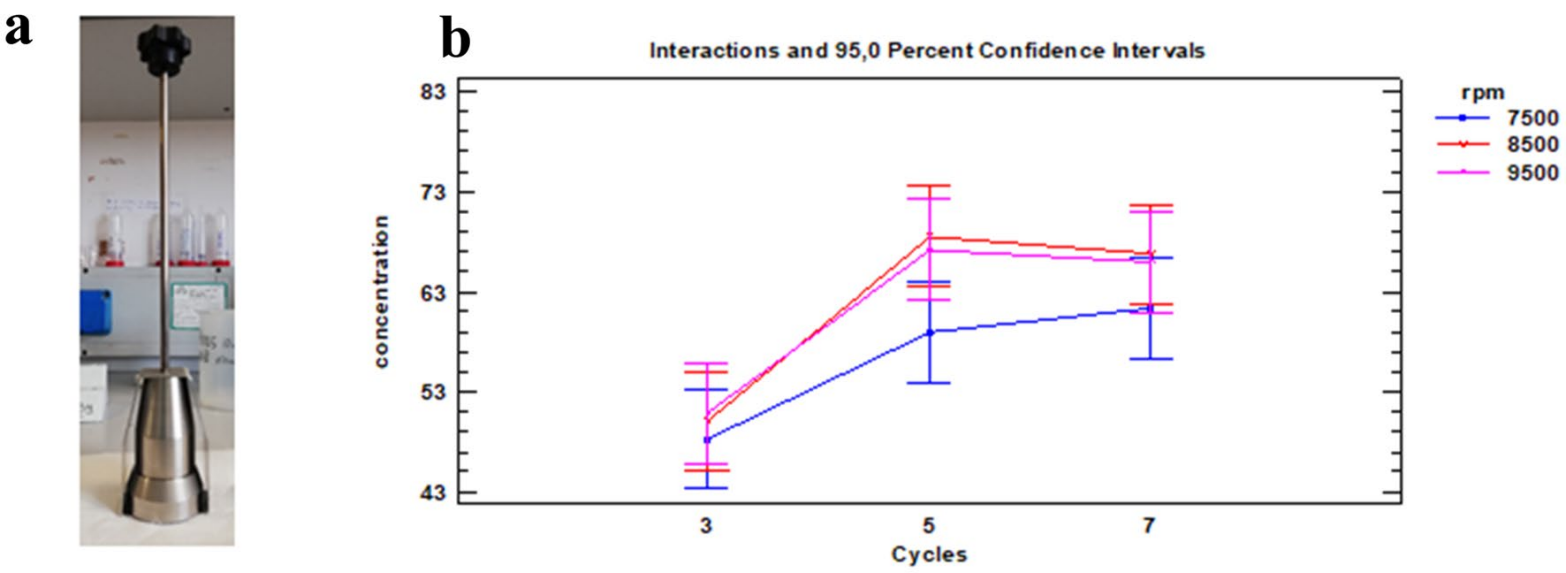
(**a**) Home-made closed stainless-steel mortar; (**b**) Two-way ANOVA interaction plot for homogenization step. Concentration values expressed as µg protein/mg tissue.

The study initially aimed to develop a high-throughput, quick and simple procedure for extracting proteins from aliquots of skin tissue to be identified by MS/MS. The coriaceous nature of full-thickness skin, which includes subcutaneous fat, dermis, and epidermis, makes protein extraction particularly challenging due to high lipid content, insolubility, and extensive protein cross-linking^23^. A first attempt to pulverize a small amount (about 100 mg) of skin samples frozen in liquid N_2_ with conventional mortar and pestle failed, as it led to inefficient pulverization. A closed stainless-steel mortar that could be fully immersed in liquid N_2_ was therefore designed and manufactured. It consists of a mortar, a pestle, and a sleeve that adhere closely together and are quick to assemble and clean (Fig. 1a). Following pulverization, bead-beating homogenization was investigated by using beads of 2.8 µm, the size recommended by the manufacturer to process hard tissues. By comparing the results obtained using steel (MK28-R) and ceramic (CK28-R) beads, it was found that the total protein concentration extracted was not significantly different (*p* > 0.05; n = 3); steel beads were chosen for the study. Different homogenization conditions were tested by varying the number of cycles (3, 5, 7) and rotation speed (7500, 8500, 9500 rpm) with fixed cycle duration (30 s) and inter-cycle waiting time (40 s). A two-way analysis of variance with interaction estimated that, at a confidence level of 95%, only the two main factors (i.e., number of cycles and rotation speed) were statistically significant, whereas the interaction term was not significant. From the interaction plot (Fig. 1b) it was observed that at 5 cycles and 8500 rpm the protein concentration stabilized at about 68 µg protein/mg tissue, without significant differences at higher cycle numbers and rpm. Thus, these homogenization conditions were chosen for all subsequent experiments. The precision in terms of total protein concentration was calculated as intra-individual and inter-individual RSD%, obtaining values lower than 10% (n = 3) and 35% (n = 26), respectively.

### Mass spectrometry-based proteomic analysis of pooled samples

After optimization of the extraction protocol, a high-throughput shotgun proteomic strategy based on a nanoLC-HRMS/MS approach was developed for the proteomic profiling of extracts from small fragments of cadaveric ecchymotic (E) and normal (N, control sample) skin. The developed analytical platform permitted the identification of about 2000 proteins for each sample; the number of identified proteins was comparable to that obtained by Bliss et al. by exploiting cryosectioning of skin tissues and 2D-LC separation^23^. The achieved high proteome coverage is strictly related to the efficiency of protein extraction combined with the sensitivity performance provided by nanoflow-LC chromatographic separations.

### Evaluation of the precision of the protein extraction method for nanoLC-HRMS/MS-based proteomic analysis of skin specimens

In order to evaluate the intermediate precision of the protein extraction protocol and nanoLC-HRMS/MS analysis, we compared the samples 1N, 2N, 1E, and 2E with the corresponding independent replicates 4N, 5N, 4E, and 5E, which were obtained from the same individuals, repeating the entire workflow procedure of protein extraction and nanoLC-HRMS/MS analysis one month later. The results clearly show that the protein extraction procedure has a good intermediate precision in terms of LFQ signal intensity and the number of identified proteins, which never differed by more than 2.5% and 6%, respectively (Table 2). The Pearson correlation graphs (Supplementary Fig. S1) and coefficient values (Table 2)—0.98 and 0.95 for the comparisons between the replicates of ecchymotic skin and 0.97 and 0.98 for normal skin—reflect the high intermediate precision of the extraction method.

**Table 2:** Number of identified proteins, sum of the LFQ intensity signal and Pearson correlation value between extraction replicates.

Sample	Number of identified proteins	Sum LFQ signal intensity	Pearson correlation
1E	1833	44,059	0.98
4E	1796	43,301	
2E	1756	42,356	0.95
5E	1773	42,835	
1N	2054	48,992	0.97
4N	2002	47,695	
2N	1873	44,828	0.98
5N	1975	46,891	

### Proteome profiling of ecchymotic skin

A shotgun LFQ proteomic approach was applied to investigate the proteome profile of ecchymotic and normal skin of cases. A Principal Component Analysis (PCA) was carried out by grouping quantitative data of proteins in the ecchymotic (1E, 2E, 3E, 4E, 5E) and normal groups (1N, 2N, 3N, 4N, 5N) (Supplementary Fig. S2), suggesting a differential clustering of the two types of skin samples.

**Figure 2:**
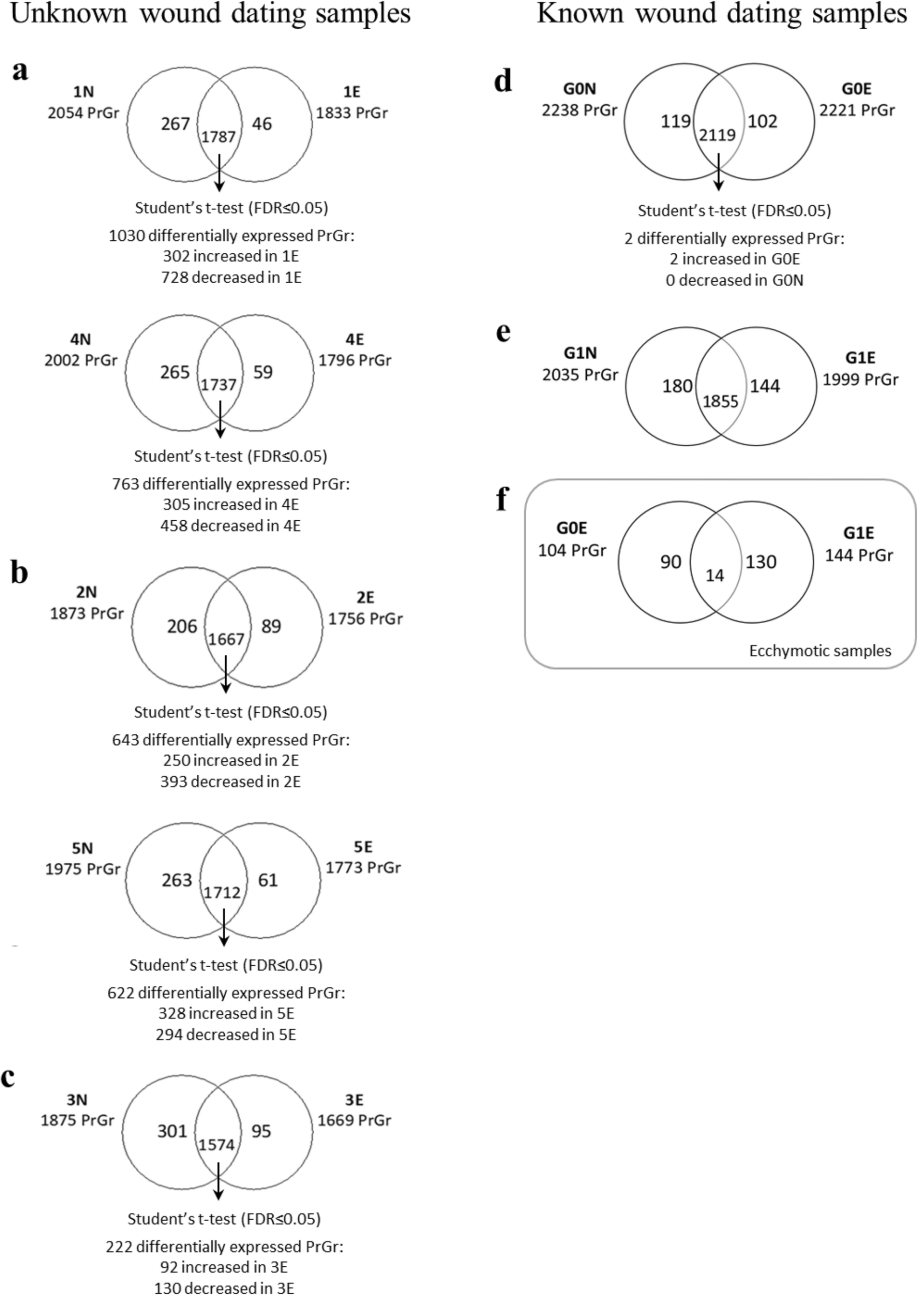
Venn diagrams of proteins identified in ecchymotic (E) and normal (N) skin in the comparisons: (**a**) 1E versus 1N and 4E versus 4N replicates; (**b**) 2E versus 2N and 5E versus 5N replicates; (**c**) 3E versus 3N; (**d**) G0E versus G0N; (**e**) G1E versus G1N; (**f**) G0E versus G1E. A statistical evaluation was applied for each comparison, where applicable. Proteins were considered to be differentially expressed if they were present only in one condition or showed significant t-test difference (Student’s t-test FDR ≤ 0.05).

The pairwise comparison between the proteomic analysis of ecchymotic tissue pools with the corresponding normal skin pools allowed us to identify the proteins in common and those that are present only in either one of the skin types. For each comparison a Student’s t-test (FDR ≤ 0.05) was carried out to identify proteins differentially present among the different conditions, as reported in the Venn diagrams shown in Fig. 2a–c.

Once the analytical strategy was developed, sample pairs from 7 additional individuals with known wound age were analyzed. In particular, 5 individuals died within 1 h after the trauma (individuals 19–23, group G0, Table 1) and two died within 1–12 h after the injury (individuals 24 and 25, group G1, Table 1). It should be pointed out that individuals whose skin lesions predate their death of a known amount of time are rare in the forensic practice, thus limiting the number of samples at our disposal. The proteome profile of both ecchymotic and normal tissue of these samples was determined using the same workflow procedure applied for cases with unknown wound dating. Figure 2d,e shows the Venn diagrams of the comparison between ecchymotic and normal skin for these samples.

**Figure 3:**
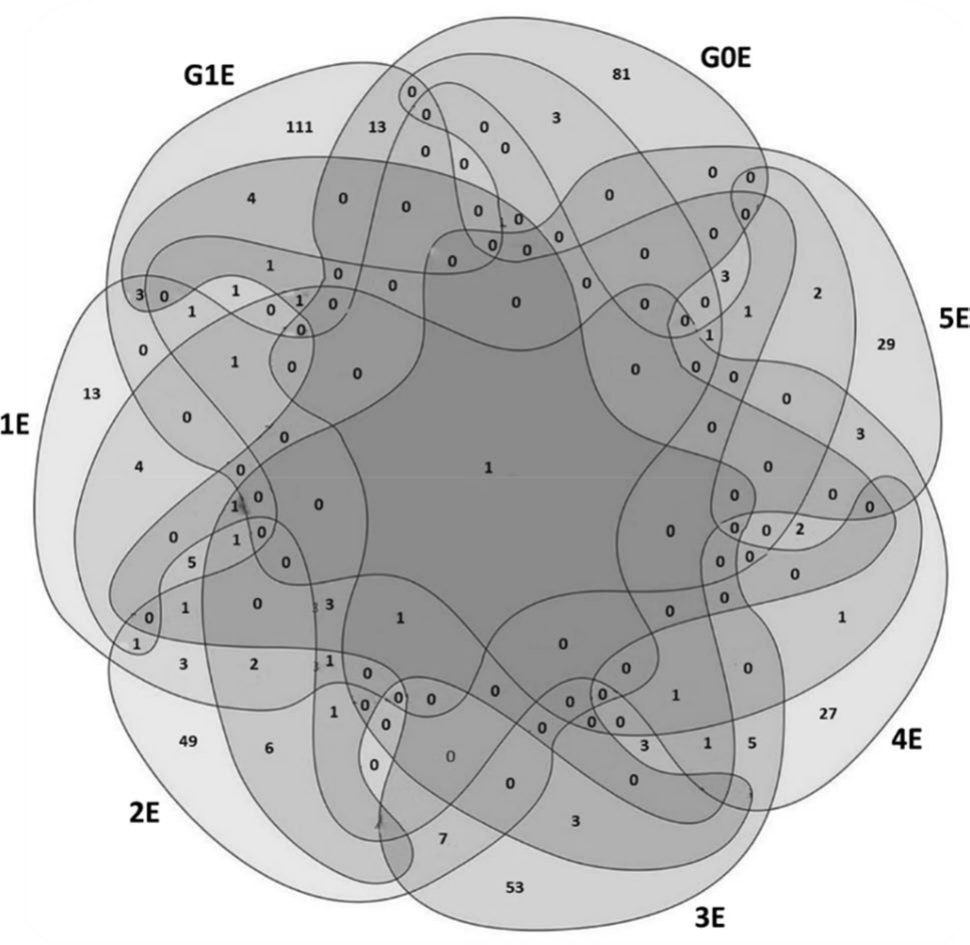
Venn diagram of the proteins identified by MS/MS exclusively in the ecchymotic skin in the comparison 1E versus 2E versus 3E versus 4E versus 5E versus G0E versus G1E.

The overall comparison between all ecchymotic samples (1E, 2E, 3E, 4E, 5E, G0E and G1E) in terms of proteins present only in the ecchymotic tissue respect to the corresponding adjacent normal skin led to the identification of a single protein, Glycophorin A (GYPA) common to all ecchymotic data sets (Fig. 3).

Glycophorins are a group of red blood cell (RBC) transmembrane proteins, described for the first time by Fairbanks et al.^29^. GYPA is the predominant member of this family, and it is considered a high-sensitive forensic marker for bleeding and, therefore, for wound vitality based on immunochemical methods. As recently discussed by Vignali et al.^4^, the most significant marker of wound vitality proved to be GYPA: it has been demonstrated that post-mortem alterations do not modify the reactivity of the GYPA, which is resistant to putrefaction for several weeks both in air and in water. Therefore, it can be extremely useful from a forensic point of view, to identify foci of vital hemorrhagic infiltration^30,31^, especially when there is no macroscopically evidence of such infiltration^32^. Although there may be some doubt as to whether the presence of red blood cells around damaged blood vessels is a certain sign of the vitality of a wound, the first microscopic step towards such a diagnosis is usually the observation of the presence of the mechanical consequence of a lesion, i.e., red blood extravasation. It may therefore be necessary to confirm the antemortem nature of a lesion by looking for molecules in the inflammatory cascade—however, observation of extravasated red blood cells is still a preliminary step in such a diagnosis.

**Figure 4:**
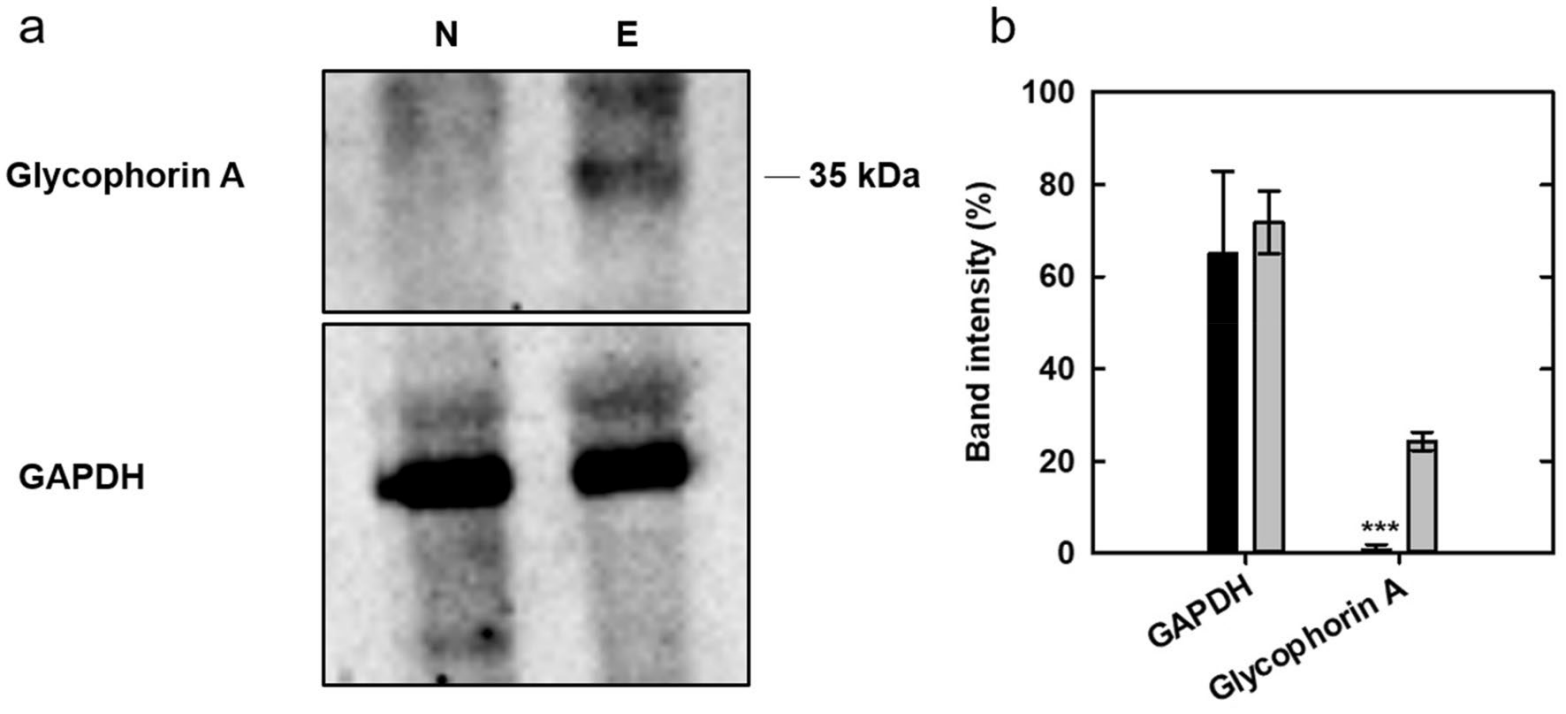
(**a**) Cropped bands of WB probed with anti-Glycophorin A antibody (upper panel) of the extracts from ecchymotic (E) and normal (N) tissue pools from individuals 13–18. GAPDH was used as a loading control (lower panel) on the same samples. Original full-length blot images and both replicates used for the analysis are provided in Supplementary Fig. S3. (**b**) Densitometric analysis of the WB in the extracts from ecchymotic (grey) and normal (black) tissue pools. Data are mean ± standard deviation from two different gels. ***p < 0.001 ecchymotic versus normal skin.

The presence of GYPA in both ecchymotic and adjacent normal skin of pooled samples was verified by Western blotting (WB) analysis on samples 3N and 3E, confirming that Glycophorin A is exclusively present in ecchymotic skin (Fig. 4), since GYPA gives an undetectable band with intensity at noise level in normal skin.

This finding suggests that this protein can be used as a MS-detectable biomarker of wound vitality.

### Identification of proteins in ecchymotic skin from cases with known wound dating

To assess the contribution of wound age on the skin proteome, proteins that were exclusively present in the ecchymotic skin of the G0 or G1 groups and not in the corresponding normal skin samples, or that showed a significant t-test difference (Student's t-test FDR ≤ 0.05), were compared. This comparison allowed us to identify 90 and 130 proteins exclusively expressed in G0E and G1E, respectively (Fig. 2f and Supplementary Table S1).

A PANTHER (Protein ANalysis THrough Evolutionary Relationships) analysis on these proteins revealed that they can be clustered in several pathways (Table 3). Proteins attributed to the inflammation chemokine and cytokine signaling pathway were present both in G0 and G1 samples, suggesting that their expression starts immediately after trauma and continues for at least 12 h. Cytokines involved in the healing process are a myriad and guide all the phases of wound healing, generally divided into coagulation, inflammation (with the removal of dead tissues), re-epithelialization, granulation tissue formation, angiogenesis, and scar formation.

**Table 3:** List of pathways identified by Panther classification system analysis (PANTHER software, release 17.0) for the proteins exclusively expressed in G0E and G1E.

PANTHER pathway common to G0E and G1E	Gene
*Common to G0E and G1E*	
Inflammation mediated by chemokine and cytokine signaling pathway (P00031)	MYH3, ITGA9 (for G0E); RHOG, MYH14, PAK1 (for G1E)
*In G0E only*	
Integrin signalling pathway (P00034)	ITGA9, ARF6
Wnt signaling pathway (P00057)	MYH3, PCDHGA12
Glycolysis (P00024)	ALDOA, PKLR
Huntington disease (P00029)	HIP1, ARF6, HIP1R
*In G1E only*	
EGF receptor signaling pathway (P00018)	NF1, MRAS, RHOG
Cytoskeletal regulation by Rho GTPase (P00016)	SSH3, DIAPH1, MYH14, PAK1
Angiogenesis (P00005)	PTPN11, CTNNB1, PAK1
Alzheimer disease-presenilin pathway (P00004)	CTNNB1, MLLT4
CCKR signaling map (P06959)	PTPN11, CTNNB1, PAK1

Proteins involved in cytoskeletal regulation by Rho GTPase, angiogenesis and CCKR signalling were found only in wounds that predated death by at least 1 h, suggesting that their expression does not occur immediately after the lesion. During wound repair, Rho GTPases are known to coordinate cytoskeletal response and repair mechanisms^33^, of which angiogenesis is part^34^. Despite the limited number of samples, we therefore suggest that these MS-detectable proteins are potential biomarkers for wound dating.

## Conclusions

Determining the vitality of a wound is a major challenge in forensic pathology, especially in cases of decomposed bodies. In general, several markers of wound vitality have been investigated, and progress has been made in wound-age estimation in the last few years, though results were scarcely reproducible. In the present study, an analytical workflow for highly precise proteomic analysis of full-thickness skin by nanoLC-HRMS was developed. The only protein uniquely identified in all ecchymotic samples was GYPA, which was validated by Western blot analysis. This finding is in accordance with literature studies based on immunochemical assays, thus strengthening the evidence that GYPA can be considered a MS-detectable marker of vital ecchymosis. As reported in the literature, GYPA appears to be resistant over time after death, making it a useful marker in corpses with advanced putrefaction phenomena.

The application of the analytical protocol to ecchymotic skin samples of known age relative to death permitted to identify other proteins differentially expressed in ecchymotic samples, although further analysis on larger datasets will be required for their validation.

The present study confirmed that mass spectrometry-based proteomics is a valuable tool for reaching conclusions in forensic death investigations; the devised sample treatment protocol could be the basis for the development of a target LC-MS/MS strategy for biomarker determination in skin tissues.

## Supplementary Information

Supplementary Information 1.

Supplementary Information 2.

## Data Availability

The mass spectrometry proteomics data are publicly available: they have been deposited in the PRIDE partner repository for the ProteomeXchange Consortium (https://www.ebi.ac.uk/pride/login) with identifier: PXD033677.
