# Supplementary material for: Mass spectrometry-based proteomic strategy for ecchymotic skin examination in forensic pathology
Source: Sci Rep. 2023 Apr 14;13:6116. doi: 10.1038/s41598-023-32520-9 (PMC10104867; doi:10.1038/s41598-023-32520-9)
Supplement: Supplementary file 1 — Supplementary Information 1. [file 41598_2023_32520_MOESM1_ESM.docx]

**Supplementary Materials**

**Mass spectrometry-based proteomic strategy for ecchymotic skin examination in forensic pathology**

Lorenzo Toma^1,‡^, Giulia Vignali^2,^^‡^, Elisa Maffioli^3,‡^, Stefano Tambuzzi^2^, Roberta Giaccari^4^, Monica Mattarozzi^1,^*, Simona Nonnis^3,5,^*, Marco Milioli^6^, Lorenzo Franceschetti^2^, Gianluca Paredi^4^, Armando Negri^3^, Benedetta Riccardi^6^, Cristina Cattaneo^2^, Maria Careri^1^, Gabriella Tedeschi^3,5^, Stefano Bruno^4^

^1^Department of Chemistry, Life Sciences and Environmental Sustainability, University of Parma, 43124 Parma, Italy

^2^Legal Medicine Institute, Department of Biomedical Sciences for Health, University of Milan, 20133 Milan, Italy

^3^Department of Veterinary Medicine and Animal Science, University of Milan, 26900 Lodi, Italy

^4^Food and Drug Department, University of Parma, 43124 Parma, Italy

^5^CRC Innovation for Well-Being and Environment (I-WE) University of Milan, 20133 Milan, Italy

^6^Department of Pharmacokinetic, Biochemistry and Metabolism, Global Research and Preclinical Development, Chiesi Farmaceutici Spa, 43122 Parma, Italy

^‡^ Co-first authors. They have contributed equally to the work.

* Corresponding authors:

Monica Mattarozzi, [monica.mattarozzi@unipr.it](mailto:monica.mattarozzi@unipr.it); ORCID id: 0000-0002-6766-4616

Simona Nonnis, [simona.nonnis@unimi.it](mailto:simona.nonnis@unimi.it); ORCID id: 0000-0002-3453-7282


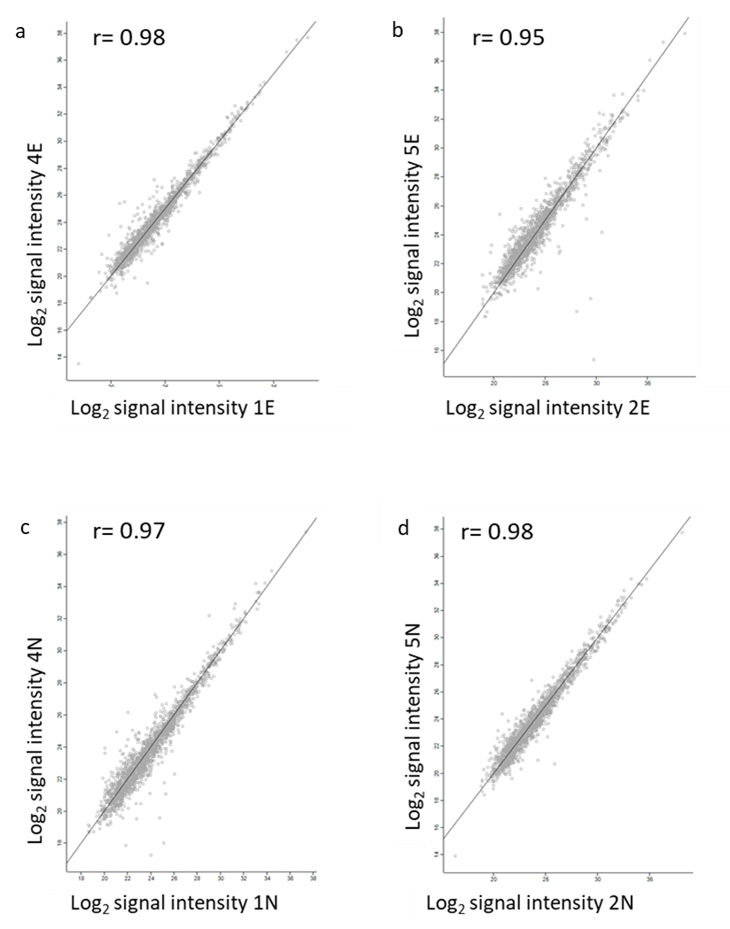


**Figure S1.** Scatter plots depicting the correlation between the first and the second protein extraction replicates in comparisons: (a) 1E vs 4E; (b) 2E vs 5E; (c) 1N vs 4N; (d) 2N vs 5N.


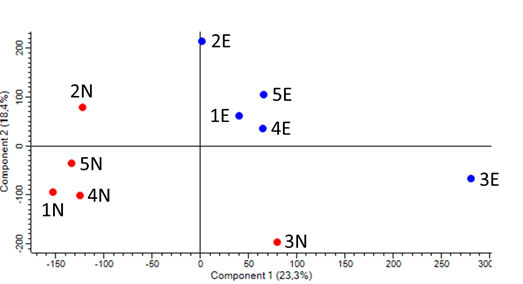


**Figure S2.** A representative Principal Component Analysis of ecchymotic (blue) and normal (red) skin specimens of cases with unknown wound dating.


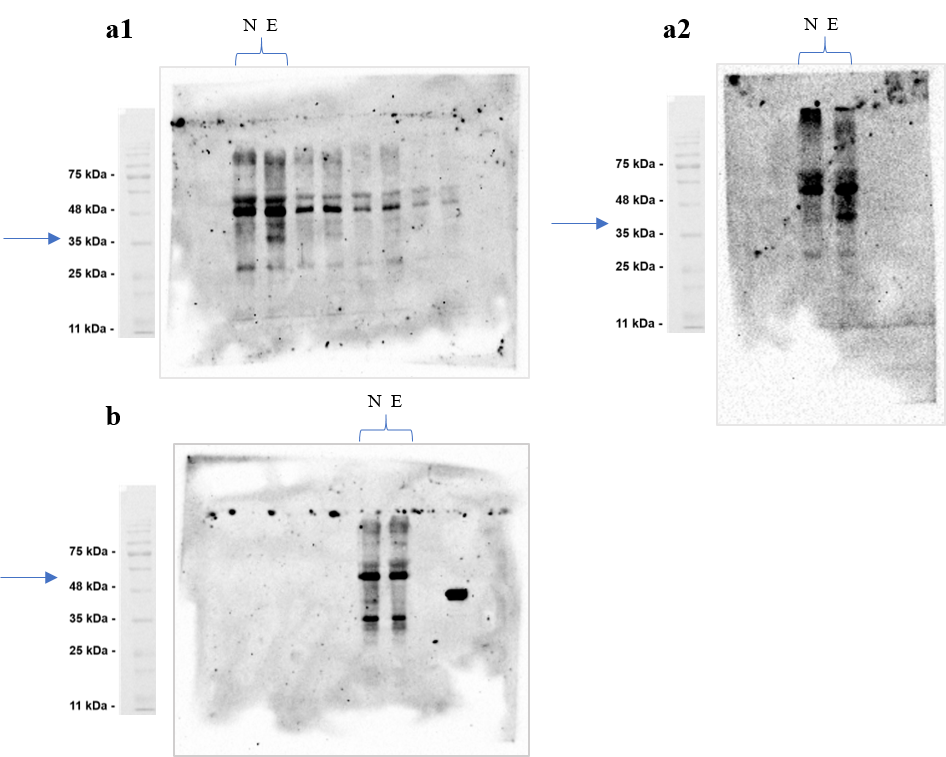


**Figure S3.** (a) Full-length WB images probed with anti-Glycophorin A antibody in replicate (a1, a2) on extracts of ecchymotic (E) and normal (N) tissue pools from individuals 13 to 18. (b) GAPDH was used as a loading control and processed in parallel in two different gels\WBs. The relevant lanes are indicated with braces and the relevant bands are indicated with arrows. The relevant lanes and bands are reported in Figure 4 of the manuscript. The primary antibodies were rabbit anti-CD235a (Glycophorin A) (Thermo Fisher Scientific, PA5-85882, 1:1000) and rabbit anti-GAPDH (Sigma Aldrich, HPA040067, 1:2500). The anti CD235a antibody also reacted with a protein of unknown identity at around 50 kDa. The identity of the Glycophorin A band was determined based on its MW at around 35 kDa. MWs were assessed using the MW marker from Applichem (protein marker VI) and visualized by Coomassie staining on the same gels later processed for WB analysis. The edges of membranes are highlighted in gray. The full SDS-PAGE gels were blotted and probed with the antibodies.
